# The Association of Unfairness with Mental and Physical Health in a Multiethnic Sample of Adults: Cross-sectional Study

**DOI:** 10.2196/26622

**Published:** 2021-05-10

**Authors:** Ken Resnicow, Minal Patel, Molly Green, Alyssa Smith, Elizabeth Bacon, Stefanie Goodell, Dylan Kilby, Madiha Tariq, Asraa Alhawli, Nadia Syed, Jennifer Griggs, Matthew Stiffler

**Affiliations:** 1 Department of Health Behavior & Health Education University of Michigan School of Public Health Ann Arbor, MI United States; 2 Center for Health Communications Research Rogel Cancer Center University of Michigan Ann Arbor, MI United States; 3 Rogel Cancer Center University of Michigan Ann Arbor, MI United States; 4 Arab Community Center for Economic and Social Services Dearborn, MI United States

**Keywords:** unfairness, discrimination, health disparities, social determinants, substance use, mental health, physical health, disparity, ethnicity, health outcome, behavior, outcome, cross-sectional, survey

## Abstract

**Background:**

Two psychosocial constructs that have shown consistent associations with negative health outcomes are discrimination and perceived unfairness.

**Objective:**

The current analyses report the effects of discrimination and unfairness on medical, psychological, and behavioral outcomes from a recent cross-sectional survey conducted in a multiethnic sample of adults in Michigan.

**Methods:**

A cross-section survey was collected using multiple approaches: community settings, telephone-listed sample, and online panel. Unfairness was assessed with a single-item previously used in the Whitehall study, and everyday discrimination was assessed with the Williams 9-item scale. Outcomes included mental health symptoms, past-month cigarette use, past-month alcohol use, past-month marijuana use, lifetime pain medication use, and self-reported medical history.

**Results:**

A total of 2238 usable surveys were collected. In bivariate analyses, higher unfairness values were significantly associated with lower educational attainment, lower age, lower household income, and being unmarried. The highest unfairness values were observed for African American and multiracial respondents followed by Middle Eastern or North African participants. Unfairness was significantly related to worse mental health functioning, net adjustment for sociodemographic variables, and everyday discrimination. Unfairness was also related to self-reported history of depression and high blood pressure although, after including everyday discrimination in the model, only the association with depression remained significant. Unfairness was significantly related to 30-day marijuana use, 30-day cigarette use, and lifetime opiate use.

**Conclusions:**

Our findings of a generally harmful effect of perceived unfairness on health are consistent with prior studies. Perceived unfairness may be one of the psychological pathways through which discrimination negatively impacts health. Future studies examining the relationships we observed using longitudinal data and including more objective measures of behavior and health status are needed to confirm and extend our findings.

## Introduction

There has been considerable interest on the part of researchers and policy makers in understanding the social, economic, psychological, and behavioral factors that account for the many health disparities that are evident in the United States and the world [1-5]. Although socioeconomic factors are the most commonly examined drivers of disparities, social and interpersonal factors have also received considerable attention [3,6-9]. Two psychosocial constructs that have shown consistent associations with health outcomes are discrimination and perceived unfairness. The former has been extensively examined in both the United States [3,9-13] and globally [8,9,14], while studies of unfairness and health have been limited to a handful of studies in the United Kingdom and the Netherlands [15-17]. No studies have reported the joint effects of these 2 constructs.

In perhaps the first published study on unfairness, DeVogli et al [16] used the prospective data of 8298 individuals from The Whitehall II study, who were civil servants originally ages 35-55 at the study onset in 1985 through 1998. Baseline unfairness data were collected between 1991 and 1993 (during phase 3 of the study), and respondents were tracked for health outcomes on average 11 years through 2003 to 2004 (phase 7 of the study). The authors found that their single-item unfairness measure was associated with higher odds of clinically verified heart disease as well as worse physical and mental functioning, with the latter being assessed with the 36-Item Short Form Survey (SF-36) questionnaire [18,19] adjusted for sociodemographic and other risk factors. Unfairness was also positively related with being female, obese, and less physically active. Higher unfairness was associated with higher cigarette use but also higher rates of alcohol abstinence.

In a second study [15], again based on the Whitehall study, phase 3 data on unfairness were used to predict cardiovascular risk factors, over the subsequent 6 years through phase 5. The authors found that baseline unfairness was significantly associated with higher rates of low serum high-density lipoprotein, high serum triglycerides, hypertension, high fasting blood glucose, and elevated waist circumference.

A third study, conducted in the Netherlands, used a 9-item perception of unfairness scale, administered in 2008 [17]. Of those completing this unfairness questionnaire, 1282 adults also completed the SF-36 [18,19] in 2003, 2008, and 2010, which was used to classify respondents as experiencing either physical or mental health decline between 2003 and 2010 and between 2008 and 2010, with unfairness scores from 2008 being used as the predictor. In general, higher scores on unfairness were associated with significantly higher odds of both physical and mental health decline. Higher scores on the unfairness measure were also associated with lower socioeconomic position (eg, a composite of income and education).

The health effects of discrimination have been reported in hundreds of studies. One measure of discrimination that has been particularly popular is the Everyday Discrimination Scale developed by Williams and others [3,10,11]. The Everyday Discrimination Scale focuses on what are sometimes referred to as microaggressions, smaller acts of discrimination, racism, or prejudice; for example, the scale includes items such as “people act as if you are inferior,” “you are treated with less respect than others,” and “people act as if you are dishonest.” Everyday discrimination is distinguished from more major experiences of discrimination, such as being unfairly fired from a job, maltreated by the police, or denied a bank loan.

The everyday discrimination scale has been associated with a wide range of adverse mental and physical health outcomes including depression, anxiety, distress [8,9,12-14], and overall well-being [9,13], breast cancer among women under the age of 50 years [20], and high blood pressure, although the relationship with the latter is often conditional on other variables [9]. Some studies have used unfair treatment as the measure of discrimination [21], although the 2 constructs likely represent different psychological and social phenomena [22]. One distinction between the 2 constructs is that everyday discrimination measures the occurrence of events, whereas unfairness can be thought of as more related to the perception of such events. Additionally, while discrimination measures multiple potential events, unfairness in the Whitehall studies and in the current investigation was measured with a single global item.

No study has reported the relationship of both discrimination and unfairness with either mental or physical health outcomes. The current analyses from a recent cross-sectional survey conducted in a multiethnic sample of adults in the state of Michigan provide insight into the effects of discrimination and unfairness on medical, psychological, and behavioral outcomes.

## Methods

### Measures

#### Independent Variables

##### Unfairness

Unfairness was assessed with a single-item from the Whitehall study [15,16], which was worded as follows: ‘‘I often have the feeling that I am being treated unfairly.’’ Participants rated their response on a 6-point scale (1, strongly disagree; 2, moderately disagree; 3, slightly disagree; 4, slightly agree; 5, moderately agree; and 6, strongly agree). We analyzed unfairness both as a continuous variable and as a categorical variable recoded into 3 levels low (responses 1 and 2), medium (responses 3 and 4), and high (responses 5 and 6) to facilitate data presentation.

##### Discrimination

We used the 9-item Everyday Discrimination scale developed by Williams [3,11]. Respondents indicated how often they experience 9 types of discrimination with responses ranging from “Never” to “Almost Everyday.” We computed a mean across the 9 items, which resulted in a score range of 1-6. Respondents were required to answer at least 5 of the 9 items to be included in the analyses.

#### Dependent Variables

Mental health symptoms were assessed with the Patient Health Questionnaire-4 (PHQ-4) [23] which asks how often over the past 2 weeks participants have experienced the following problems: little interest or pleasure in doing things; feeling down, depressed, or hopeless; feeling nervous, anxious, or on edge; and not being able to stop or control worrying. All items were answered with a 1-4 scale, with 4 being “not at all” and 1 being “nearly every day”. Higher scores are indicative of better mental health status. The Cronbach α for the 4 items in our sample was .91.

Health behaviors assessed included current cigarette use, defined as consuming at least 100 cigarettes all time and having smoked on at least some days in the past month [24]; past month alcohol, defined as having consumed at least one drink of any alcoholic beverage at least once in the past 30 days [24]; past month marijuana, defined as any use in the past 30 days; lifetime pain medication, which was queried with an item from the 2017 Youth Risk Behavior Survey [25]: “During your life, how many times have you taken prescription pain medicine without a doctor’s prescription or differently than how a doctor told you to use it? (Count drugs such as codeine, Vicodin, OxyContin, Hydrocodone, and Percocet.)” Use was considered more than 2 times in one’s lifetime.

Self-reported medical history was assessed by asking if the respondent had ever been diagnosed with cancer; diabetes or high blood sugar; high blood pressure or hypertension; depression; or a heart condition, such as heart attack, angina, or congestive heart failure. Each variable was answered with 0 (no) or 1 (yes).

Demographic variables assessed included age (collapsed into 4 groups: 18-35, 30-45, 45-65, and >65 years), household income (collapsed into 4 groups: under US $10,000, US $10,000 to US $49,999, US $50,000 to US $99,999, and >US $100,000), education (collapsed into 4 groups: high school or lower, some college, college graduate, graduate school or higher), gender, country of birth (United States vs not United States), and marital status (no or yes). These are presented in Table 1.

**Table 1:** Sample demographics (N=2238).

Variable		Respondents, n (%)
**Gender**		
	Male	726 (32.50)
	Female	1508 (67.50)
**Education**		
	High school or lower	558 (25.04)
	Some college	690 (30.97)
	College graduate	659 (29.58)
	Graduate school	321 (14.41)
**Age (years)**		
	18-35	566 (25.76)
	30-45	493 (22.44)
	45-65	881 (40.10)
	> 65	257 (11.70)
**Income (US$)**		
	Under $10,000	227 (10.79)
	$10,000 to $49,999	887 (42.18)
	$50,000 to $99,999	611 (29.05)
	$>100,000	378 (17.97)
**Born in the United States**		
	Yes	1902 (85.29)
	No	328 (14.71)
**Race**		
	White	1183 (53.00)
	African American	525 (23.52)
	Middle Eastern	403 (18.06)
	Multiracial	105 (4.70)
	Other	16 (0.72)
**Marital status**		
	Married/living as married	1134 (50.90)
	Not married	1094 (49.10)
**Modality**		
	Online	1415 (63.23)
	Telephone	496 (22.16)
	Paper	327 (14.61)

### Procedures

Surveys were collected in 2019 using multiple methodologies including community settings, telephone, and online panel. The community sample was a convenience sample, whereas the telephone and online sample was built to match the demographic representation of the University of Michigan Rogel Cancer Center catchment area.

#### Community Administration for Middle Eastern and North African Participants

The Middle Eastern and North African (MENA) sample was collected at community settings across 3 Michigan counties that included 2 supermarkets frequented by the MENA community, 1 health clinic serving a predominantly Arab American population, 1 health clinic serving a predominantly Chaldean population, a state university with a high number of Arab American students, 4 mosques with a high proportion of Yemeni and Lebanese worshippers, 2 Chaldean churches, and a recreation center frequented by Lebanese youth.

Participants were given the option of completing surveys using pen and paper or online forms (tablet provided), with or without assistance, in English or Arabic. For those opting to complete surveys at home, we provided a self-addressed stamped envelopes or a web address to complete the online version. Both paper and electronic surveys required active consent and testament that the respondent was over 18 and self-identified as Arab or Chaldean. Data collectors, many of whom were fluent in both English and Arabic, were trained in interviewing by study staff. Participants received a US $25 gift card after completing their survey. A total of 406 participants were accrued through this method, 87 of whom completed their survey in Arabic.

#### Community Administration for White and African American Participants

We distributed surveys at 5 community-based educational events sponsored by the University of Michigan Rogel Cancer Center. Participants were able to complete surveys using printed forms or online with provided tablets, and they received a US $25 gift card after completing their survey. A total of 214 participants were accrued through this method.

#### Telephone

The telephone survey, conducted by Harris Interactive Inc, used a quota sample to reach 501 adults who indicated that they were aged 18 years or over, living in zip codes serviced by the University of Michigan Rogel Cancer Center, and self-identified as either White/Caucasian or Black/African American. The survey, conducted in English, averaged about 23 minutes and consisted of 44 substantive questions and 18 demographic questions. The survey was fielded from May 1, 20219, to July 9, 2019.

Known landline and cell phone numbers were obtained from Dynata, formerly Survey Sampling International. Numbers were randomly selected from within identified zip codes. We oversampled (with a target of at least 40% of the final sample) African American participants by selecting telephone exchanges that were estimated to have an at least 50% African American population.

A maximum of 8 contacts were attempted for each number: the initial dialing attempt plus up to 7 subsequent dialing attempts. A US $10 incentive was offered to the survey participants. The overall response rate was 9%: 8% for landline numbers and 9% from cell phone numbers. A total of 496 participants were accrued through this method, approximately half through landline and half through cell phone.

#### Online

A quota sample was recruited via an online panel through a commercial survey research organization (Dynata), which maintains a demographically diverse web panel of people who opt in to taking selected surveys. Panel members who log on to Dynata’s site are routed (in a randomized fashion) to available surveys based on their demographic characteristics and needs of open surveys We provided specific county level quotas for individuals aged 18-80 years in 40 Michigan counties served by the University of Michigan Rogel Cancer Center. We oversampled African American participants so that they would comprise at least 20% of the sample. Additional details about Dynata can be found at their website [26]. A total of 1122 participants were accrued through this method. It is possible that participants could have appeared in both the telephone and online panels; however, given the anonymous nature of the data collection and the fact they were conducted independently, we cannot determine how many might have been duplicated. Based on the number of participants available in the targeted zip codes and the number that completed the surveys, the likelihood is small.

### Analyses

We first present mean unfairness and discrimination values by sociodemographic variables (Table 2). For mental health symptoms, we present (labeled Model 1) linear regression results for the association of unfairness (trichotomized into low, medium, and high to facilitate data presentation) with mental health symptoms, adjusting for income, education, age, gender, and marital status (this is Model 1 referenced in Table 3, Table 4, and Table 5). We then present results (labeled Model 2) with discrimination added to the regression model (this is Model 2 referenced in Table 3, Table 4, and Table 5).

For health behaviors and medical history, we first report multivariate logistic regression (ORs and 95% CIs), using unfairness (trichotomized) as the primary independent variable, adjusting for income, education, age, gender, and marital status, and then we report a model adding discrimination (labeled Model 2).

## Results

A total of 2238 usable surveys were collected, of which two-thirds (n=1508) were completed by females. A little less than half (980/2228, 43.79%) had college or higher educational attainment, and about half were aged 45 or higher, married, and reported a household income above US $50,000 per year. Most, (1902/2230, 85.29%), were born in the United States. About half (1183/2232, 53.00%) were White, 23.52% (2232/2238) were African American, 18.06% (403/2232) were MENA, and 4.70% (105/2232) were multiracial.

As shown in Table 2, the mean value for the unfairness item was 2.62 (SD 1.61), with a range of 1-6; meanwhile, the mean for everyday discrimination was 1.87 (SD 1.06) with a range of 1-6. The 2 variables were moderately correlated (*r*=0.54; *P*<.001).

In bivariate analyses, higher unfairness values were significantly associated with lower educational attainment, lower age, lower household income, and being unmarried. Birthplace was unrelated to unfairness. For race, the highest values were observed for African American and multiracial respondents followed by MENA respondents. White respondents reported the lowest values, and they were significantly lower than African American, multiracial, and MENA respondents in pairwise comparisons.

**Table 2:** Mean scores for unfairness and everyday discrimination by demographic variables.

Variable			Unfairness, mean (SD)	Everyday discrimination, mean (SD)
**Gender**				
	Male	2.62 (1.66)		1.88 (1.12)
	Female	2.63 (1.59)		1.87 (1.03)
**Education^a^**				
	High school or less	2.82 (1.67)^b,c^		1.97 (1.18)^b,c^
	Some college	2.76 (1.59)^d,e^		1.94 (1.06)^d,e^
	College graduate	2.42 (1.53)^b,d^		1.79 (1.03)^b,d^
	Graduate school	2.43 (1.63)^c,e^		1.72 (0.90)^c,e^
**Age^a^**				
	18-35	2.94 (1.58)^b,c,d^		2.27 (1.18)^b,c,d^
	30-45	2.71 (1.61)^b,e,f^		2.05 (1.17)^b,e,f^
	45-65	2.45 (1.58)^c,e^		1.66 (0.89)^c,e,g^
	>65	2.31 (1.63)^d,f^		1.40 (0.70)^d,f,g^
**Income^a^ (US $)**				
	Under 10,000	2.97 (1.68)^b,c^		2.12 (1.24)^b,c,d^
	10,000 to 49,999	2.85 (1.62)^d,e^		1.95 (1.07)^b,e,f^
	50,000 to 99,999	2.43 (1.51)^b,d^		1.81 (0.97)^c,e^
	>100,000	2.26 (1.55)^c,e^		1.75 (1.08)^d,f^
**Born in the United States^h^**				
	Yes	2.62 (1.61)		1.92 (1.08)
	No	2.68 (1.61)		1.56 (0.89)
**Race**				
	White	2.25 (1.48)^b,c,d^		1.75 (0.97)^b,c^
	African American	3.27 (1.67)^b,e^		2.12 (1.15)^b,d,e^
	Middle Eastern	2.73 (1.58)^c,e,f^		1.69 (0.97)^d,f^
	Multiracial	3.20 (1.69)^d,f^		2.55 (1.36)^c,e,f^
	Other	2.75 (1.39)		2.17 (1.04)
**Marital status^a^**				
	Married/living as married	2.38 (1.56)^b^		1.72 (0.97)^b^
	Not married	2.87 (1.62)^b^		2.03 (1.12)^b^
Total			2.62 (1.61)	1.87 (1.06)

^a^Group means differ *P*<.01.

^b-g^Rows with common superscript differ *P*<.05

^h^Group means in everyday discrimination differ *P*<.01.

As shown in Table 3, unfairness was significantly related to mental health symptoms, after adjustment for sociodemographic variables (Model 1), with higher unfairness values associated with worse (lower scores) mental health status. In addition to an overall significant effect, all pairwise contrasts were significant. The association, both overall and pairwise, remained significant after inclusion of everyday discrimination scores in the regression model (Model 2). The *R*^2^ increased from 0.17 to 0.22 when everyday discrimination was added to the model.

**Table 3:** Adjusted least squares mean mental health symptoms by unfairness and discrimination^a^.

Model values			Mental health symptoms, mean (SE)
**Model 1^b,c^**			
	Low unfairness	13.70 (0.23)^d,e^	
	Medium unfairness	12.45 (0.23)^d,f^	
	High unfairness	10.80 (0.30)^e,f^	
**Model 2^c,g^**			
	Low unfairness	13.43 (0.22)^d,e^	
	Medium unfairness	12.84 (0.24)^d,f^	
	High unfairness	11.70 (0.30)^e,f^	

^a^Lower scores indicate worse mental health status.

^b^Model 1: adjusted for age, gender, race, income, education, and marital status; *R*^2^=0.17.

^c^Group means differ *P*<.01.

^d-f^Rows with common superscript differ *P*<.05.

^g^Model 2: adjusted for age, gender, race, income, education, marital status, and everyday discrimination; *R*^2^=0.22.

As shown in Table 4, unfairness was significantly related to self-reported history of depression and high blood pressure, after adjustment for sociodemographic variables (Model 1). Higher unfairness values were significantly associated with higher odds of depression, when both the middle and high groups were compared to the lowest group, and for high blood pressure, when the highest group was compared to the lowest group. After inclusion of everyday discrimination scores in the model (Model 2), there was still an overall association with depression, and the pairwise contrast of highest to lowest remained significant. In Model 2, the effect of unfairness on high blood pressure disappeared. The addition of everyday discrimination to the model increased the *R*^2^ value by 0.01% to 0.02%.

**Table 4:** Adjusted odds of self-reported history of illness by unfairness and discrimination^a^.

Predictor			History of depression^b^	History of diabetes		History of heart disease		History of high blood pressure		History of cancer
**Model 1^c^ unfairness (*R*^2^)**			0.12	0.09		0.05		0.20		0.06
	Low	REF^d^		REF	REF		REF		REF	
	Middle	*1.57 (1.24-1.98)* ^e^		1.21 (0.92-1.58)	1.01 (0.69-1.48)		1.11 (0.87-1.42)		1.18 (0.82-1.69)	
	High	*2.93 (2.18-3.95)*		1.03 (0.72-1.45)	1.31 (0.84-2.06)		*1.49 (1.08-2.05)*		1.14 (0.71-1.82)	
**Model 2^f^ unfairness (*R*^2^)**			0.14	0.10		0.07		0.21		0.07
	Low	REF		REF	REF		REF		REF	
	Middle	1.19 (0.92-1.54)		1.15 (0.82-1.54)	0.80 (0.52-1.22)		1.00 (0.761-1.309)		0.99 (0.67-1.48)	
	High	*1.89 (1.35-2.64)*		0.84 (0.57-1.234)	0.87 (0.52-1.45)		1.22 (0.85-1.74)		0.78 (0.46-1.32)	

^a^Unless otherwise stated, the data are reported as OR (95% CI).

^b^Overall variable *P*<.01.

^c^Model 1: adjusted for age, gender, race, income, education, and marital status.

^d^REF: reference group.

^e^Italicized CIs indicate statistically significant ORs.

^f^Model 2: adjusted for age, gender, race, income, education, marital status, and everyday discrimination.

As shown in Table 5, unfairness was significantly related to 30-day marijuana use, 30-day cigarette use, and lifetime opiate use, after adjustment for sociodemographic variables (Model 1). There was no association with 30-day alcohol use. Specifically, higher unfairness values, when both the middle and highest groups were compared to the lowest group, were associated with significantly higher odds of cigarette and opiate use, whereas for marijuana, the contrast was significant only for the highest compared to the lowest group. When everyday discrimination scores were added to the model (model 2), the effect on marijuana was no longer significant and the effect on cigarettes was only significant for the comparison of the middle to the lowest group. In addition, the effect on alcohol use became significant. However, the effect was in the opposite direction than with the other substances. That is, higher unfairness was significantly associated with lower odds of 30-day alcohol use. The addition of everyday discrimination to the model increased the *R*^2^ value by 0.01% to 0.02%.

**Table 5:** Adjusted odds of self-reported substance use by unfairness^a^.

Predictor		30-day alcohol	30-day marijuana	30-day cigarettes	Lifetime opiates
**Model 1^b^ unfairness (*R*^2^)**		0.14	0.08	0.05	0.07
	Low	REF^c^	REF	REF	REF
	Middle	0.82 (0.64-1.04)	1.14 (0.82-1.59)^d^	*1.69 (1.28-2.23)* ^e,f^	*1.37 (1.07-1.74)* ^d^
	High	0.76 (0.54-1.07)	*1.74 (1.17-2.57)* ^d^	*1.96 (1.39-2.76)* ^f^	*1.41 (1.03-1.92)* ^d^
**Model 2^g^ unfairness (*R*^2^)**		0.14^f^	0.09	0.08	0.08
	Low	REF	REF	REF	REF
	Middle	*0.73 (0.56-0.96)* ^d^	0.89 (0.62-1.28)	*1.38 (1.01-1.87)*	1.00 (0.76-1.30)
	High	*0.65 (0.45-0.95)* ^d^	1.22 (0.79-1.89)	1.42 (0.972-2.10)	0.84 (0.59-1.19)

^a^Unless otherwise stated, the data are reported as OR (95% CI).

^b^Model 1: adjusted for age, gender, race, income, education, and marital status.

^c^REF: reference group.

^d^*P*<.05.

^e^Italicized CIs indicate statistically significant ORs.

^f^*P*<.01.

^g^Model 2: adjusted for age, gender, race, income, education, marital status, and everyday discrimination.

## Discussion

Unfairness was significantly related to worse mental health functioning, net adjustment for sociodemographic variables, and everyday discrimination. Unfairness was related to self-reported history of depression and high blood pressure after an adjustment for sociodemographic variables was made although after including everyday discrimination in the model, only the association with depression remained significant.

Unfairness was also significantly related to 30-day marijuana use, 30-day cigarette use, and lifetime opiate use. Adding everyday discrimination scores to the model attenuated these effects, with the association with marijuana no longer being significant and the effect on cigarettes only being significant for the comparison of the middle group to the lowest group.

Our findings of a generally harmful effect of perceived unfairness on health are consistent with prior studies [15-17]. Perceived unfairness, although moderately correlated with everyday discrimination, appears to serve as a unique predictor of health status and health behavior. Alternatively, given that, in some instances, adding everyday discrimination to the model attenuated the impact of unfairness on outcomes, unfairness may also serve as a partial mediator of discrimination; that is, it may be one of the psychological pathways through which discrimination negatively impacts health: it may be a consequence of discrimination. On the other hand, adding discrimination to the model (except for mental health) generally only increased the *R*^2^ value by 1% or 2%, suggesting that unfairness captures most of the same variance in health outcomes as does discrimination.

In our study, we found that higher unfairness was associated with lower educational attainment, lower age, lower household income, and being unmarried. The higher rates of unfairness observed for individuals with lower education and income is consistent with both UK [15,16] and Dutch studies [17]. Age was unrelated to unfairness in the Whitehall study [16], which differs from our findings. We found no differences by gender, which is inconsistent with the Whitehall study, where women had higher levels of reported unfairness [16].

With regard to race, we found the highest unfairness values were observed among African American and multiracial respondents, followed by MENA respondents. White respondents reported the lowest values, and they were significantly lower than African American, multiracial, and MENA respondents in pairwise comparisons. Prior studies did not report race effects, as their samples were more homogeneous than that of this study. The Whitehall study sample, for example, consisted of 90% White and 5% South Asian participants [27].

We found that the effect of unfairness on 30-day alcohol was in the opposite direction to the other substances we examined. That is, higher unfairness was associated with lower odds of 30-day alcohol use. The protective effect of unfairness on alcohol intake was not entirely unexpected. A few previous studies have found that higher levels of unfairness and discrimination were not associated with increased alcohol use [9,16,28]. In our study, this relationship was in part confounded by religiosity, as more religious respondents were both more likely to abstain from alcohol and to report higher levels of unfairness (data not shown). Controlling for religion reduced the protective effect of unfairness on alcohol use.

The study has several limitations. First, the data were all cross sectional, which limits our ability to determine causation; reverse causality cannot be excluded. For example, higher rates of illness or substance use could drive higher perceptions of unfairness or discrimination, rather than the inverse.

Secondly, unfairness may be caused by everyday discrimination. Experiencing discrimination could lead one to perceive life as unfair. Given the 2 variables were moderately correlated (*r*=0.54) and that the effects of unfairness on health outcomes generally remained after adjustment for discrimination, it appears that unfairness may function as a unique contributor to health outcomes, above that of discrimination. It should also be noted that while the 9-item everyday discrimination scale assesses various types of discriminatory behaviors, the single-item unfairness measure we used only assesses global perceptions. Perhaps more granular assessment of unfairness, more akin to how discrimination is typically assessed, would yield more robust findings.

Furthermore, illness outcomes were all self-reported, which likely increased the error of these measures. Given this, the analyses might have underestimated the association of unfairness on health, unless they share a common reporting bias, which then would have spuriously inflated their association. Finally, our sample was recruited in Michigan, so the generalizability of our findings to other populations is unclear.

Our findings have implications for both research and practice. With regard to research, our results indicate that unfairness should be included as a potential variable in studies of health and health disparities in addition to the standard set of socioeconomic and social indicators. Unfairness may also be more amenable to intervention than discrimination and other disparity drivers. Because unfairness relates more to the perception or perseveration of events rather than simply their occurrence, cognitive interventions that help individuals cope with these perceptions may merit investigation.

## Abbreviations

MENA: Middle Eastern and North African
PHQ-4: Patient Health Questionnaire-4
SF-36: 36-Item Short Form Survey
